# *Candida vulturna* Outbreak Caused by Cluster of Multidrug-Resistant Strains, China

**DOI:** 10.3201/eid2907.230254

**Published:** 2023-07

**Authors:** Han Du, Jian Bing, Xiaohong Xu, Qiushi Zheng, Tianren Hu, Yajuan Hao, Shuping Li, Clarissa J. Nobile, Ping Zhan, Guanghua Huang

**Affiliations:** Huashan Hospital, Fudan University, Shanghai, China (H. Du, J. Bing, Q. Zheng, T. Hu, G. Huang);; Sinopharm Tongmei General Hospital, Datong, China (X. Xu, Y. Hao, S. Li);; University of California, Merced, California, USA (C.J. Nobile);; Affiliated Hospital of Jiangxi University of Chinese Medicine, Nanchang, China (P. Zhan)

**Keywords:** *Candida vulturna*, emerging fungal pathogen, fungi, multidrug resistance, antimicrobial resistance, adhesion, biofilm formation, China

## Abstract

*Candida vulturna* belongs to the *Candida haemulonii* species complex and is phylogenetically related to *C. auris.* We report a *C. vulturna* outbreak among persons in Shanxi Province, China, during 2019–2022. Isolates were resistant to multiple antifungal drugs and exhibited enhanced adhesion and biofilm formation properties.

*Candida vulturna*, a fungal pathogen that is phylogenetically related to *C. haemulonii* and *C. auris,* was isolated from flowers in a taxonomic study of yeasts in 2016 (*1*,*2*). Since then, *C. vulturna* has been sporadically isolated in different countries from clinical specimens such as blood, wounds, and peripherally inserted central catheters (PICCs) (*1*–*4*). *C. vulturna*, *C. haemulonii*, and *C. auris* belong to the *Metschnikowia*/*Candida* clade (*1*,*5*). Antifungal drug resistance, especially to the azoles, is a common feature of species within this clade. During 2009–2022, fungal infections caused by the reportedly rare species *C. haemulonii* and *C. auris* have become more prevalent in clinical settings (*1*,*6*–*10*). The increased occurrence of those infections could be the result of the widespread use of antifungal agents in clinical and agricultural settings, as well as the environmental changes caused by human activities (*10*–*12*). 

In China, reports of infections caused by the superbug fungus *C. auris* have been relatively infrequent; however, the prevalence of *C. haemulonii* and associated species in the *C. haemulonii* complex has been steadily increasing in recent years (*8*,*13*). For our study, we analyzed deidentified health records of patients infected with *C. vulturna*, as approved by the ethics committee of a general hospital in Shanxi Province, China.

## The Study

We selected a total of 19 patients, 17 male and 2 female, who had been infected with *C. vulturna* during January 1, 2019–October 26, 2022 (Appendix Figure 1). We isolated 16 *C. vulturna* strains directly from the blood through venipuncture and 7 strains from a PICC line tip of the 19 patients (Appendix Table). We initially identified the strains as *C. haemulonii* complex species by growth on CHROMagar *Candida* medium (CHROMagar, https://www.chromagar.com) and confirmed by sequencing of the ribosomal internal transcribed spacer (ITS) region. Most cases were identified in 2019; *C. vulturna* infections were identified in 2 patients during January 1, 2020–January 1, 2022. Enhanced hygiene measures taken at that time may have dampened the spread of *C. vulturna* in the hospital.

**Figure 1:**
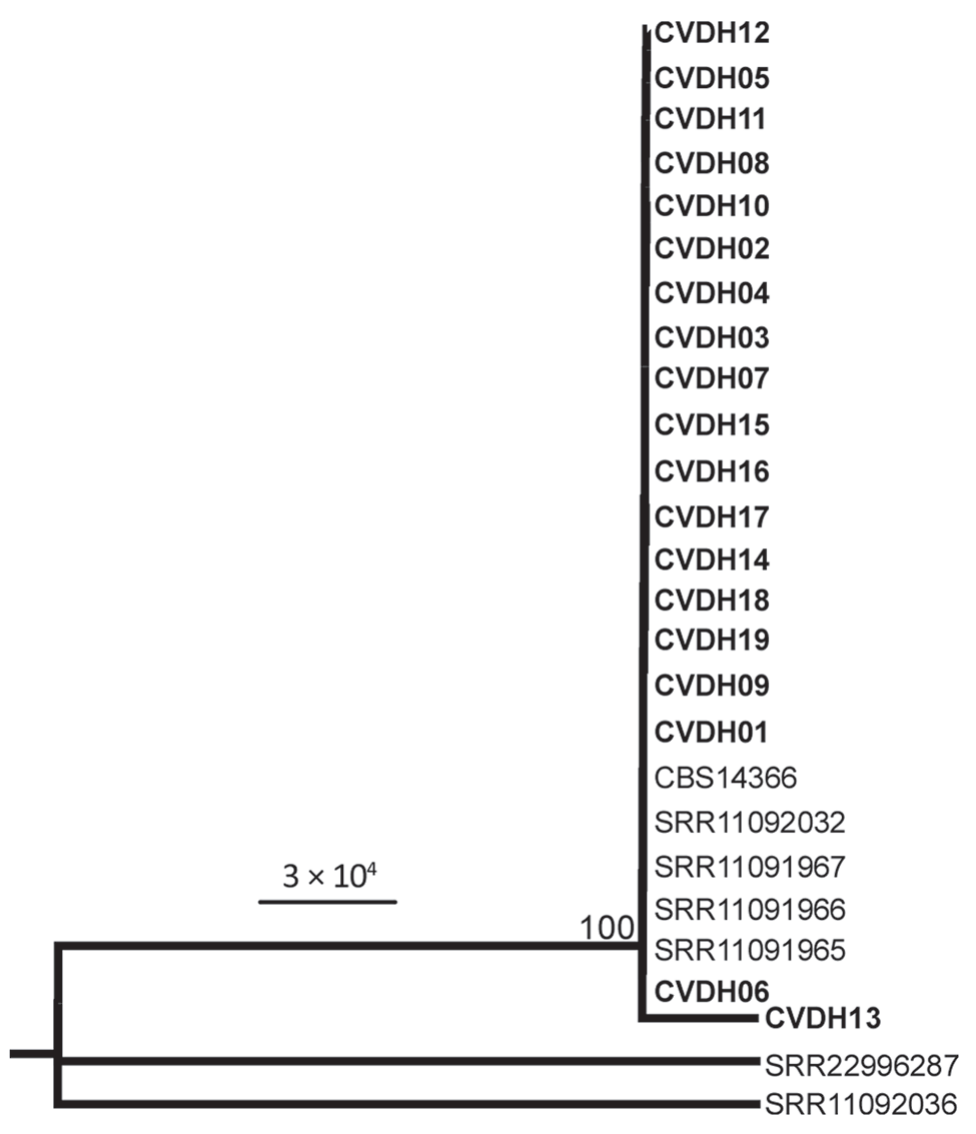
Maximum-likelihood phylogeny analysis of *Candida vulturna* strains from 19 infected patients in Shanxi Province, China, January 1, 2019–October 26, 2022, based on multilocus sequence typing (MLST). Eight genes (*AAT1, ACC1, ADP1, ALA1, ERG11, RPB1, RPB2,* and *ZWF1*) were concatenated and used for phylogenetic analyses. The tree was generated using the program RAxML (https://cme.h-its.org/exelixis/web/software/raxml). The general time reversible model, gamma distribution, 1,000 bootstraps, and midpoint root were adopted. Bold text indicates strains isolated in this study; reference strain data from whole-genome sequencing is from the National Center for Biotechnology Information gene database (accession nos. SRR11091965–67, SRR11092032, SRR11092036, SRR22996287). Sequences for strain CBS14366 were retrieved from its genomic assembly (GenBank accession no. GCA_026585945.1). Strains CVDH01-CVDH19 were isolated from patients of *C. vulturna* infection (cases C1–C19; Table; Appendix Figure 1). Scale bar indicates substitutions per site.

On the basis of results of the ITS and multilocus sequence typing for 8 conserved genes, we then performed phylogenetic analyses on the isolates. All strains isolated in this study (CVDH01–19) were closely related by phylogenetic analyses and clustered together in 1 clade (Figure 1; Appendix Figure 2).

The hospital has 1 intensive care unit (ICU). Of the 19 patients we identified as infected with *C. vulturna*, 11 were from the ICU, 4 were from the neuroscience ward, and 4 were from other departments within the hospital. The age range of patients was 13–83 years (median 63 years). Because all patients had PICC lines for delivery of medications and *C. vulturna* strains were isolated from the PICC line tips of 7 patients, the use of PICC lines could be a major risk factor for *C. vulturna* infection. Other risk factors could include traumatic injuries, hypertension, cancer, and blood and pulmonary infections (Appendix Table). We also conducted environmental screening assays but were unable to detect or isolate *C. vulturna* from hospital surfaces, including walls, floors, bedside tables, bed sheets, bed frames, blood pressure cuffs, and chairs.

We used 1 representative *C. vulturna* strain from each patient for subsequent antifungal drug susceptibility testing and phenotypic analyses (Appendix Table). Using the breakpoints established for *C. albicans*, we determined that all 19 of the *C. vulturna* strains tested were resistant to azole drugs (Table). All isolates were resistant to amphotericin B (MIC 4 mg/L) but were susceptible to echinocandins (MICs <0.125 for caspofungin, <0.125 for anidulafungin, <0.5 for micafungin), and flucytosine (MIC 0.06).

**Table:** Susceptibility profiles of *Candida vulturna* isolates from 19 infected patients to 9 antifungal drugs, Shanxi Province, China, January 1, 2019–October 26, 2022*

Patient no.	Strain ID	FLC	VOC	ITC	POC	CAS	MFG	AFG	5-FC	AMB
C1	CVDH01	**32**	**32**	**64**	**64**	0.125	0.5	0.125	0.06	**4**
C2	CVDH02	**128**	**32**	**32**	**16**	0.06	0.5	0.125	0.06	**4**
C3	CVDH03	**64**	**32**	**32**	**32**	0.06	0.25	0.125	0.06	**4**
C4	CVDH04	**128**	**32**	**16**	**16**	0.125	0.5	0.06	0.06	**4**
C5	CVDH05	**128**	**32**	**32**	**32**	0.125	0.5	0.06	0.06	**4**
C6	CVDH06	**128**	**32**	**32**	**32**	0.125	0.5	0.125	0.06	**4**
C7	CVDH07	**256**	**64**	**64**	**64**	0.06	0.25	0.125	0.06	**4**
C8	CVDH08	**128**	**32**	**32**	**32**	0.25	0.5	0.125	0.06	**4**
C9	CVDH09	**128**	**32**	**32**	**64**	0.06	0.25	0.25	0.06	**4**
C10	CVDH10	**128**	**32**	**16**	**16**	0.125	0.5	0.125	0.06	**4**
C11	CVDH11	**256**	**64**	**64**	**64**	0.125	0.5	0.125	0.06	**4**
C12	CVDH12	**128**	**32**	**64**	**64**	0.06	0.25	0.125	0.06	**4**
C13	CVDH13	**64**	**32**	**32**	**32**	0.06	0.25	0.125	0.06	**4**
C14	CVDH14	**64**	**16**	**32**	**32**	0.03	0.5	0.03	0.06	**4**
C15	CVDH15	**128**	**64**	**32**	**16**	0.06	0.25	0.125	0.06	**4**
C16	CVDH16	**128**	**32**	**64**	**32**	0.125	0.5	0.125	0.06	**4**
C17	CVDH17	**64**	**32**	**32**	**32**	0.06	0.5	0.06	0.06	**4**
C18	CVDH18	**64**	**8**	**32**	**32**	0.06	0.5	0.06	0.06	**4**
C19	CVDH19	**64**	**32**	**32**	**32**	0.06	0.5	0.06	0.06	**4**

*MIC assays were performed according to Clinical and Laboratory Standards Institute microdilution guidelines. Bold text indicates antifungal resistance (based on the breakpoints for *C. albicans*). AFG, anidulafungin; AMB, amphotericin B; CAS, caspofungin; FLC, fluconazole; ITC, itraconazole; MFG, micafungin; POC, posaconazole; VOC, voriconazole; 5-FC, flucytosine.

**Figure 2:**
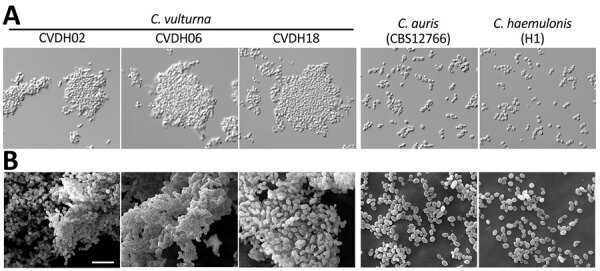
Morphologies of 3 representative *C. vulturna* isolates from 19 infected patients in Shanxi Province, China, January 1, 2019–October 26, 2022. *C. auris* (CBS12766) and *C. haemulonii* (H1) served as reference strains. A) Adhesion phenotypes of *C. vulturna* isolates grown in liquid Lee’s glucose medium at 30°C for 24 h. Strains CVDH02, CVDH06, and CVDH18 exhibited strong adhesiveness, whereas the *C. auris* and *C. haemulonii* reference strains grew as separate single cells under the same culture conditions. B) Biofilm formation of *C. vulturna* isolates. *C. auris* (CBS12766) and *C. haemulonii* (G7) served as reference strains. Biofilms were developed on silicone squares at 30°C for 48 h. Lee’s glucose medium was used for biofilm growth. Scale bar indicates 10 µm. Morphologies for the other 16 *C. vulturna* isolates and 2 *C. auris* strains are shown in Appendix Figure 3.

When grown in liquid media, we observed that the cells from the *C. vulturna* (CVDH) strains isolated in this study formed large aggregates and exhibited enhanced adhesion and biofilm formation abilities. This feature was similar to that of *C. auris* strain SJ01, which formed enhanced biofilms under both in vitro and in vivo conditions (*14*). (Figure 2; Appendix Figure 3).

## Conclusion

A serious threat to human health is the emergence of new multidrug-resistant fungal species. Both the widespread use of antifungal agents and the reduced susceptibility of these emerging species to antifungal drugs could contribute to the epidemiologic shifts toward multidrug-resistant fungal pathogens that we are increasingly observing in clinical settings. In this study, we report an outbreak of *C. vulturna*, which is phylogenetically closely related to *C. haemulonii* and *C. auris*, in a general hospital in Shanxi Province, China. We observed that the implementation of general enhanced hygiene measures remarkably decreased overall infection rates during the COVID-19 pandemic period (January 1, 2020–January 1, 2022) in this hospital; our findings suggest that the transmission of *C. vulturna* may be preventable through enhanced disinfection methods. Most of the *C. vulturna* isolates we obtained were from patients with bloodstream infections, defined as a single isolation of *C. vulturna* from blood obtained through venipuncture. Phylogenetic analyses indicated that the outbreak strains were closely related (Figure 1; Appendix Figure 2), implying that those strains could have originated from the same ancestor.

Striking characteristics of the *C. vulturna* strains isolated in this study were their enhanced adhesion and biofilm formation abilities. It is conceivable that those characteristics may be key contributors in promoting the spread of *C. vulturna* strains between patients during this outbreak. Consistent with this hypothesis, we observed that the use of PICC lines was a critical risk factor for *C. vulturna* infections. Another notable characteristic of the *C. vulturna* strains isolated in this study was their reduced susceptibilities to azole drugs and amphotericin B (Table), which has also been observed in other species of the *C. haemulonii* complex (*6*,*7*,*13*).

The occurrence of infections caused by fungal species of the *Metschnikowia* clade has become more and more frequent in clinical settings, especially during 2009–2022 (*1*,*6*,*8*,*13*). The widespread use of antifungal drugs in clinical settings and fungicides in agricultural settings could be contributors to the increased emergence of these multidrug resistant fungal pathogens. Given the transmissible, adhesive, and antifungal drug–resistant characteristics of emerging *C. vulturna* clinical isolates, *C. vulturna* could be a serious upcoming threat to hospital infections worldwide.

Appendix Additional information about *Candida vulturna* outbreak, China.
