# Supplementary material for: Candida vulturna Outbreak Caused by Cluster of Multidrug-Resistant Strains, China
Source: Emerg Infect Dis. 2023 Jul;29(7):1425–8. doi: 10.3201/eid2907.230254 (PMC10310381; doi:10.3201/eid2907.230254)
Supplement: Appendix — Additional information about Candida vulturna outbreak, China. [file 23-0254-Techapp-s1.pdf]

Article DOI: https://doi.org/10.3201/eid2907.230254

EID cannot ensure accessibility for supplementary materials supplied by authors. Readers who have difficulty accessing supplementary content should contact the authors for assistance.

Candida vulturna Outbreak Caused by Cluster of Multidrug-Resistant Strains, China

Han Du, Jian Bing, Xiaohong Xu, Qiushi Zheng, Tianren Hu, Yajuan Hao, Shuping Li, Clarissa J. Nobile, Ping Zhan, Guanghua Huang

Appendix

Additional Case Details

Case 4 was initially admitted to the department of geriatrics; Case 6 the department of general surgery; Cases 7 and 10 the department of neurosurgery; and Case 8 the department of orthopedics. These patients were later transferred to the ICU of the hospital. The earliest two *C. vulturna* infection cases were identified in the neuroscience ward, we suspect that *C. vulturna* was transmitted from other wards to the ICU.

Some patients were initially admitted to the general surgery, neuroscience, or other wards of the hospital prior to transfer to the ICU. Since the earliest two *C. vulturna* infection cases were identified in the neuroscience ward, we suspect that *C. vulturna* was transmitted from other wards to the ICU.

There could be multiple reasons for the reduction of infection cases during COVID. First, during COVID, the disinfectant with an increased concentration of hypochlorite (two-fold) was

used for floor disinfection. Second, disinfectants (such as 75% alcohol in the form of sprays and wipes) were available for all visitors and healthcare staff throughout the hospital. Third, the general and ICU wards strictly limited visitors.

Materials and methods

Strains and culture conditions

C. vulturna, *C. auris*, and *C. haemuloni* strains were routinely grown on solid YPD medium (2% Glucose, 2% peptone, 1% yeast extract, 2% agar). Modified Lee's glucose media (1) was used for *Candida* aggregation and biofilm assays.

For growth on nutrient agar, approximately 150 cells were plated on Lee's glucose medium and cultured at 30°C or 37°C for 3 days. For liquid culture, fungal cells were inoculated into 3 mL Lee's glucose liquid medium to an OD_{600} of 0.2 and incubated at 30°C or 37°C with shaking for 24 hours.

Environmental screening assays were performed to isolate *C. vulturna* from hospital surfaces, including walls, floors, bedside tables, bed sheets, bed rails, bed frames, blood-pressure cuffs, and chairs. More than 300 environmental samples were analyzed. Swab and wipe samples were used for culture assays on CHROMagar *Candida* medium.

To develop biofilms on silicone squares, approximately 2 x 10^6 cells of each strain were inoculated into each well containing one silicone square (10 mm x 10 mm, Bentec Medical, INC., Woodland) and 600 µL Lee's glucose medium. After incubation for 48 hours at 30°C with shaking, the silicone squares were washed gently with ddH_20 three times and used for scanning electron microscopy (SEM) assays.

SEM assays

SEM assays were performed as described in our previous publication (2). Briefly, the silicone squares with *Candida* biofilms were fixed with 2.5% glutaraldehyde. The samples were

dehydrated in increasing concentrations of ethanol (25%-50%-70%-90%-100%), followed by tert-butyl alcohol solvent displacement through a series of increasing concentration of tert-butyl alcohol (25%-50%-70%-90%-100%) and freeze-dried. Finally, the samples were coated with gold and imaged using a scanning electron microscope (FlexSEM 1000 II, HITACHI).

Antifungal drug susceptibility assays

Minimum inhibitory concentrations (MICs) were determined according to the CLSI (Clinical Laboratory Standards Institute, 2012) guidelines. Liquid RPMI-1640 medium (w/v, 1.04% RPMI-1640, 3.45% MOPs, pH was adjusted to 7.0) containing a series of concentrations of different antifungal drugs was used. MICs were determined after 24 hours incubation at 35°C. *Candida krusei* ATCC 6258 and *Candida parapsilosis* ATCC 22019 served as quality controls.

ITS- and MLST-based species identification and phylogenetic analysis

C. vulturna strains were streaked and grown on YPD plates. Genomic DNA of single colonies was extracted for PCR analysis. A fragment containing the internal transcribed spacer (ITS), partial 18S small subunit (SSU), and 28S large subunit (LSU) ribosomal sequences were amplified using the primer pair ITS1 (5'-TCCGTAGGTGAACCTGCGG-3') / NL4 (5'-GGTCCGTGTTTCAAGACGG-3'). Eight genes (*AAT1, ACC1, ADP1, ALA1, ERG11, RPB1, RPB2,* and *ZWF1*) were chosen for MLST analysis based on prior studies (3, 4). The following primers were used in the PCR reactions:

AAT1 (540 bp):

AAT1F: aaggagtacacgggtatcac, AAT1R: aacgagctcgttcaatcttc

ACC1 (515 bp):

CvACC1F: accaacaacaacaactacgc, CvACC1R: ccagccaacttcatgatgaa

ADP1 (499 bp):

ADP1F: ttcaaaaagaccaccacgag, ADP1R: acactctccaccggtaatgt

ALA1 (530 bp):

ALA1F: tgcgaactcccaaaagtgta, ALA1R: tttcaaaacccataccggtg

ERG11 (562 bp):

ERG11F: aactctcgtttgatggagca, ERG11R: aatgcaacaagaaccaagca

RPB1 (516 bp):

RPB1F: agaagagatttaatgcggtg, RPB1R: ccatgtatgtagcaacgtga

RPB2 (513 bp):

RPB2F: atcgaggagaaggtggagaa, RPB2R: ttcctcaacacagggcttca

ZWF1 (503 bp):

ZWF1F: actcgtctatcctgaaggtg, CvZWF1R: tctcgtcgtcaaggtaggat

The PCR products were sequenced and analyzed. The homologous sequences of representative species of the CTG clade were retrieved from the NCBI GeneBank or CGD (http://www.candidagenome.org/) databases. The sequences of *C. vulturna* isolates and other CTG species were analyzed using software mafft v7.015b (5). The phylogenetic tree was generated using the programme RAxML v7.3.2 (6). The General Time Reversible (GTR) model, gamma distribution, and 1000 bootstraps were adopted.

Sequence information for strains CVDH01-CVDH19.

The internal transcribed spacer (ITS) and partial ribosomal sequences for CVDH01-CVDH19 were amplified by PCR using the primer pair ITS1 (5'-TCCGTAGGTGAACCTGCGG-3') / NL4 (5'-GGTCCGTGTTTCAAGACGG-3'). The sequences are listed below.

The ITS and partial ribosomal sequences for strains CVDH01-CVDH19 (the sequences were the same):

GCGGAAGGATCATTAAAATAACACTTACACACTGATTTTGACTAGTAAATAA CCCACCAGTTAAGTTCAATTACACAATTAGTAAAACTTTCAACAACGGATCTCTTGG TTCTCGCATCGATGAAGAACGCAGCGAAATGCGATACGTAGTATGACTTGCAGACG TGAATCATCGAATCTTTGAACGCACATTGCGCCTTGGAGCATTCTCCAAGGCATGCC TGTTTGAGCGTGATTTCTTCTCACCGCACCCGGTGGTTTTGCATCCGCGCTAAATATC ATTCCAGCAGCGAAGTCTACGCTTTCACTGCTCCATGCTATTTCCTCAAATCAGGTA GGACTACCCGCTGAACTTAAGCATATCAATAAGCGGAGGAAAAGAAACCAACAGGG ATTGCCTCAGTAACGGCGAGTGAAGCGGCAAGAGCTCAACTTTGGAATCGCTCCGG CGAGTTGTAGTCTGGAGGCGGCCGGTCCGCCTTGCGCAACCAAATCTAAGTCCTCTG GAACGAGGCGCTTGAAAGGGTGACAGCCCCGTGGATTTGTCTGTTGTGCTTGGCCCC TGGTCCTGCCGACGAGTCGAGTTGTTTGGGAATGCAGCTCTAAGTGGGTGGTAAATT CCATCTAAAGCTAAATACCGGCGAGAGACCGATAGCGAACAAGTACAGTAATGGAA AGATGAAAAGCACTTTGAAAAGAGAGTGAAACAGTACGTGAAATTGTTGAAAGGGA AGGGCTTGCAGGTAGACAACTGTCAGCATCGGGTGGAGTGGAGCTAGAAGTGGGCG CTGATGTAGCAACTTCGGTTGCATTATACAACGCTCAGATAGCTCCCGTTTCGCCCG AGGATCGCCTTTTGAAGGATG

DNA sequences for MLST analysis (eight genes: *AAT1, ACC1, ADP1, ALA1, ERG11, RPB1, RPB2,* and *ZWF1*)

AAT1 sequence for strains CVDH01-CVDH19:

CGGTTCCAAGACCTACCAGGACGCGGTCAAGAACTTCATTTTCAACAACTCT GACAAGGACACCAACGGTGCTCAATTGATCAAGGACGGCCGTATTGTCACTGCCCA AACCATCTCCGGTACCGGTTCTCTCCGTGTTATCGCCGATTTCCTCAACAGATTCTAC TCCTCGGGTCAGATCATCGTTCCTAAGCCAACCTGGGCTAACCACGTCGCTGTGTTC

ACCGATGCTGGTATGAAGGCTGACTTTTACGCCTACTACGACAAGGAGAACAATGG
CTTGGACTTTGAGAATCTCAAGAAGTCTGTCGCTGCTGCTCCTGAGGAGTCTGTGAT
CTTGTTGCACGCCTGTTGCCACAACCCTACTGGTATGGACTTGACTCCCCAGGAATG
GGAGGAGGTTTTGGAGATCATCCAGCAGAAGAAGCTCTTCCCTCTTGTGGACATGGC
CTACCAGGGCTTCGCCTCCGGTAACACCTACGAGGACATTGGCTTGATCA

ACC1 sequence for strains CVDH01-CVDH19:

CAATGTCGAGTTGATTGTCGAAATCGCAGAGAGAACCAATGTCCACGCCGTG
TGGGCCGGCTGGGGCCACGCCTCGGAAAACCCCATTTTGCCCGAGATGTTGGCCGCC
CTGCCCAAAAAAATCGTGTTTATCGGCCCGCCAGGCTCCGCCATGAGGTCCTTGGGT
GACAAGATCTCCTCCACAATCGTTGCACAGCACGCCGACGTGCCCTGTATCCCCTGG
TCCGGTACGGGCGTGCTGGACGTTGAAATTGACAACGAAACGAAATTGGTCTCGGT
GTCCGAAGAGACTTACGCCAAGGGCTGCTGCACGAGTCCGGAAGACGGCTTGGAAA
AAGCCCGCCAGATCGGTTTCCCCGTCATGATCAAGGCCTCCGAGGGTGGAGGCGGT
AAAGGTATCCGTAAGGTCGACAACGAGGACGATTTTATCTCCTTGTACAAGCAGGCT
GCTAACGAGATCCCTGGCTCTCCAATT

ADP1 sequence for strains CVDH01-12 and CVDH 14-19:

GCAACGAGACGTCATGTTCGTGTGACGACGGTTGGGACGGAATTAACTGTAA
CATTTGTACAGATGATTCTGTTTGTGATGCTTTTATGCCCGAGGGTCTCAAGGGAGTC
TGTTACCAGCGAGGAGTTGTCATCAACGAGATTCACCAAATGTGCAATGTGACCAAT
CCCAAGATTATCAAGATTTTAGAGGGTGAGATTCCCCAAGCCACCTTTAGATGTGAC
AAAAAGAACAATACTTGTGATTTTCAATTCTGGATTGATGAGGTAGAATCTTTCTTTT
GTGACTTGAGTCTGTGCAAGTTTGATTACGATCTCGAGTCGAATACAACCCGCTATA
ACTGTGACAATGTGGCTTGCGAGTGTTTGCCTGGACGTATGCTTTGTGGCAAATCTG
GATCAATTGATATCTCAGAGTTCCTTGAAAAAACTATCAAGGGTCCAGGTGACTTTA
CTTGTG

ADP1 sequence for strains CVDH13:

GCAACGAGACGTCATGTTCGTGTGACGACGGTTGGGACGGAATTAACTGTAA CATTTGTACAGATGATTCTGTTTGTGATGCTTTTATGCCCGAGGGTCTCAAGGGAGTC TGTTACCAGCGAGGAGTTGTCATCAACGAGATTCACCAAATGTGCAATGTGACCAAT CCCAAGATTATCAAGATTTTAGAGGGTGAGATTCCCCAAGCCACCTTTAGATGTGAC AAAAAGAACAATACTTGTGATTTTCAATTCTGGATTGATGAGGTAGAATCTTTCTTTT GTGACTTGAGTCTGTGCAAGTTTGATTACGATCTCGAGTCGAATACAACCCGCTATA ACTGTGACAATGTGGCTTGCGAGTGTTTGCCTGGACGTATGCTTTGTGGCAAATCTG GATCAATTGATATCTCAGAGTTCCTTGAAAAAACTATCAAGGGTCCAGGTGACCTTA CTTGTG

ALA1 sequence for strains CVDH01-19:

TCAGAGCCGGTGGTAAGCACAATGACTTGGATGACGTCGGAAAGGACTCTTA TCACCACACCTTTTTCGAGATGTTGGGTAACTGGTCTTTTGGTGACTACTTCAAGAAG GAGGCTATCGAGTGGTCTTGGGAATTGTTGACTGAGGTCTTCAAGTTGGAAAAAGAC CGTTTGTACGTGACCTACTTTGAGGGTGACGAGAAGAACGGCTTGCAGCCAGACCTA GAGGCCAAGCAGTTCTGGTTGGATGTCGGTGTGGCCGAGGACCACATCTTGCCGGGT GACGCTTCTGACAACTTCTGGGAAATGGGTGATCAAGGCCCATGTGGTCCATGTTCC GAGATCCACTACGACAGAATTGGTGGAAGAAACGCCGCCCACTTGGTGAACATGGA CGACCCCAATGTTTTGGAGGTCTGGAACGTCGTGTTCATTCAGTACAACAGAGAGGC AGACTCGTCTCTTAGGCCATTGCCTAACAAGCACATTGA

ERG11 sequence for strains CVDH01-19:

GAAGAAGTTCGCAAAGACAGCCTTGACCAAGGAAGCTTTCCAAAGATACGTC CCTAGAATCCAGGAGGAGGTGTTGGACTACTTCAAGACCTGTCCTGAGTACGAGAT GAACGAGCGCAACAACGGTGTTGCCAACGTGATGAAGACCCAGCCTGAGATGACCA TCTTGACTGCTTCCAAGTCTTTGATGGGCGACGACATGAGAGCCAAGTTTGATGCCT

CTTTTGCTCAGTTGTACTCCGATTTGGACAAGGGTTTCACCCCTATCAACTTTGTTTT
CCCTCACTTGCCTTTGCCCGCTTACTGGAAGAGAGACGCTGCTCAGCAGAAGATCTC
GGCTACGTACATGTCCTTGATTAATGAGAGAAGAAGTACTGGTGACATCATCCCAGA
CAGAGACTTGATCGACTCGCTCATGACCAACTCTACCTACAAAGACGGTGTCAAGAT
GACCGACCAGGAGGTTGCCAACTTGTTGATCGGTGTCTTGATGGGTGGTCAGCACAC
TTCCGCTTCCACCTC

RBP1 sequence for strains CVDH01-19:

TGGAACGTCTGTAAGACAAAGATGGTCTGTGAAGCTGACGTCGTCAACGATG
AAGGCCAGGTTACCTCTGGAAGAGGCGGCTGTGGTCACACACAACCTACTGTTCGTA
GAGATGGTATGAAGTTGTGGGGAACATGGAAACAGAACAAACAGTTTGAGGAAAA
CGAACAGCCCGAGCGTCGTTTGTTGACCCCATCGGAGATTTTGAGTGTTTTCAGACA
CATCAGTGAAGAAGACTGTCAGAAGTTGGGCTTCAATGAAGACTATGCAAGACCAG
AGTGGATGTTGATCACCGTTCTACCTGTTCCACCCCCACCTGTGAGACCCTCGATTG
CTTTCAACGATACTGCTAGAGGTGAGGATGACTTGACATTTAAGTTGGCTGATATCA
TCAAAGCCAATATCAACGTTCAACGACTCGAAATGGACGGTTCTCCTCAGCACGTTA
TCAGTGAGTTTGAAGCTCTTTTACAGTT

RPB2 sequence for strains CVDH01-19:

TGAGGATGCCCAGACCAAGGTATTTTTGGGTAAGGTGCCTATCATGTTGCGTT
CCAAGTTCTGTATGTTGCGTGACTTGGGCGAACACGAGTTCTACGAGTTGAAGGAGT
GCCCATACGATATGGGTGGTTACTTTGTCATCAACGGTTCCGAGAAGGTTTTGATTG
CCCAGGAGCGTTCTGCTGCTAATATTGTGCAAGTCTTCAAGAAGGCGGCGCCTTCCC
CTATTTCCCACGTTGCCGAGATCAGATCCGCCCTCGAGAAGGGTTCACGGTTGATCT
CCTCCATGCAAATCAAGTTGTACGGTAGAGACGACAAGGGCACTTCCGGCAGAACC
ATCAAGGCTACCTTGCCATACATCAAAGAAGACATCCCTATCGTTATCGTTTTTAGA

GCCCTCGGTGTTGTCCCTGATGGTGATATCTTGGAGCACATTTGTTACGACGCTAAT GACTGGCAAATGTTGGAGATGT

ZWF1 sequence for strains CVDH01-19:

GAAGAGTGAGAGTCATTGTCGAGAAGCCCTTCGGCCACGATTTGGAGTCTTC CAGACAATTGCAGAAAGATTTGGCTCCTCTTTTCACTGAGGAAGAATTGTACAGAAT TGACCACTACTTGGGCAAGGAAATGGTGAAGAACTTGTTGGTGTTGCGTTTTGGTAA TGAGTTATGGTCTGGTGTGTGGAACAACAGGCATATTTCCTCGGTCCAGATTTCCTTT AAAGAGGCATTCGGAACAGAGGGAAGAGGCGGCTACTTTGACCTGATCGGCATAAT CAGAGACGTCATGCAGAACCACTTATTGCAGGTGTTGACCCTTTTGACCATGGAGAG ACCTGTGTCGTTCGACCCAGAGGCTGTGAGAGATGAAAAAGTGAAGGTGCTCAAGG CTTTTGACGATTTTAACCCCAACGACATCTTGCTCGGTCAATATGGTAAGTCTGAAG ATGGCTCTAAGCC

References

1. Huang G, Yi S, Sahni N, Daniels KJ, Srikantha T, Soll DR. N-acetylglucosamine induces white to opaque switching, a mating prerequisite in Candida albicans. PLoS Pathog. 2010;6:e1000806.
2. Du H, Guan G, Xie J, Sun Y, Tong Y, Zhang L, et al. Roles of Candida albicans Gat2, a GATA-type zinc finger transcription factor, in biofilm formation, filamentous growth and virulence. PLoS One. 2012;7:e29707.
3. Spampinato C, Leonardi D. Molecular fingerprints to identify Candida species. BioMed Res Int. 2013;2013:923742.
4. Cendejas-Bueno E, Kolecka A, Alastruey-Izquierdo A, Theelen B, Groenewald M, Kostrzewa M, et al. Reclassification of the Candida haemulonii complex as Candida haemulonii (C. haemulonii group I), C. duobushaemulonii sp. nov. (C. haemulonii group II), and C. haemulonii var. vulnera var. nov.: three multiresistant human pathogenic yeasts. J Clin Microbiol. 2012;50:3641–51.

5. Katoh K, Standley DM. MAFFT multiple sequence alignment software version 7: improvements in performance and usability. Mol Biol Evol. 2013;30:772–80.

6. Stamatakis A. RAxML-VI-HPC: maximum likelihood-based phylogenetic analyses with thousands of taxa and mixed models. Bioinformatics. 2006;22:2688–90.

Appendix Table. Detailed information on the patients with *C. vulturna* infections*

Case	Sex	Age, y	Diagnosis	Facility type	Specimen source	Strain collection date (Days after admission)	Time of admission, Days	Patient outcome
C1	Male	45	Hypertension (stage III) and Brain surgery	Neuroscience ward	Blood	Jan 17, 2019 (16 days)	Jan 1, 2019 – Feb 12, 2019 (42 days)	Routine discharge
C2	Male	60	Traumatic subarachnoid hemorrhage tSAH, intravetricular hemorrhage, scalp laceration, traumatic pneumonia	Neuroscience ward	Blood	Jun 20, 2019 (20 days)	May 31, 2019 - Sep.11, 2019 (103 days)	Routine discharge
C3	Male	57	Chronic bronchitis, pulmonary infection	ICU	Blood	Aug 3, 2019 (81 days)	May 14, 2019 – Aug 8, 2019 (86 days)	Discontinued care
C4	Male	73	Chronic cough and expectoration, Hypertension (stage III)	ICU	Blood	Aug 13, 2019 (3 days)	Aug 10, 2019 – Sep 2, 2019 (23 days)	Discontinued care
C5	Male	43	Injuries to the spleen, rib fractures	General surgery ward	Blood	Aug 16, 2019 (38 days)	Jul 9, 2019 – Nov 16, 2019 (130 days)	Routine discharge/ self care
C6	Male	78	Acute abdominal disease, Hypertension (stage I)	ICU	Blood† and PICC tip	Aug 18, 2019 (13 days)	Aug 5, 2019 – Sep 21, 2019 (47 days)	Routine discharge
C7	Female	16	Injuries to the head, face are, chest and abdominal tissues caused by car accident	ICU	Blood	Aug 27, 2019 (7 days)	Aug 20, 2019 – Dec 26, 2019 (128 days)	Routine discharge/ self care
C8	Male	73	Thoraco-abdominal and pelvic inj	ICU and Neuroscience ward	Blood	Sep 21, 2019 (14 days)	Sep 7, 2019 – Nov 6, 2019 (60 days)	Routine discharge/ self care

Case	Sex	Age, y	Diagnosis	Facility type	Specimen source	Strain collection date (Days after admission)	Time of admission, Days	Patient outcome
			uries caused by car accident					
C9	Male	46	Traumatic epidural hematoma, scalp injury and skull fracture	ICU	Blood	Oct 11, 2019 (9 days)	Oct 2, 2019 – Jan 14, 2020 (104 days)	Routine discharge/ self care
C10	Male	66	Trigeminal neuralgia, hypertension	ICU	Blood† and PICC tip	Oct 12, 2019 (73 days)	Jul 19, 2019 – Nov 14, 220 (118 days)	Routine discharge
C11	Female	13	Serious intracranial injury, multiple tissue injuries caused by car accident	ICU	Blood† and PICC tip	Nov 1, 2019 (11 days)	Oct 21, 2019 – Jan 13, 2020 (84 days)	Routine discharge
C12	Male	71	Bile duct cancer	General surgery ward	PICC tip	Nov 19, 2019 (30 days)	Oct 20, 2019 – Dec 13, 2019 (54 days)	Routine discharge
C13	Male	78	Periodic fever	General Medicine ward	Blood	Dec 28, 2019 (32 days)	Nov 26, 2019 – Jan 20, 2020 (55 days)	Routine discharge
C14	Male	83	Chronic obstructive pulmonary disease, lower respiratory tract infection, hypertension (stage III)	ICU	PICC tip	Sep 18, 2020 (19 days)	Aug 30, 2020 – Sep 22, 2019 (23 days)	Discontinued care
C15	Male	66	Paraplegia, type 2 diabetes, pulmonary and urinary tract infections	ICU	Blood	Nov 18, 2020 (19 days)	Oct 30, 2020 – Dec 15, 2020 (46 days)	Discontinued care
C16	Male	63	Consciousness Disorder, Septic shock, sepsis, pulmonary and urinary tract infections	ICU	Blood	Feb 11, 2022 (43 days)	Dec 30, 2021 – Mar 6, 2022 (66 days)	Expired
C17	Male	57	Injuries to the head caused by car accident	ICU	PICC tip	Jun 30, 2022 (35 days)	May 26, 2022 – Jul 13, 2022 (48 days)	Routine discharge

Case	Sex	Age, y	Diagnosis	Facility type	Specimen source	Strain collection date (Days after admission)	Time of admission, Days	Patient outcome
C18	Male	66	Advanced gastric cancer, bladder cancer	Internal Medicine - Oncology	Blood† and PICC tip	Jul 24, 2022 (17 days)	Jul 7, 2022 – Aug 30, 2022 (54 days)	Routine discharge
C19	Male	60	Intracranial infection	Neuroscience ward	Blood	Aug 26, 2022 (32 days)	J Jul 25, 2022 – Oct 26, 2022 (94 days)	Routine discharge

*PICC, peripherally inserted central catheter; ICU, intensive care unit.

†When *C. vulturna* was isolated from two or more specimen sources, strains isolated from the blood samples were used for biological and DNA sequencing analyses.

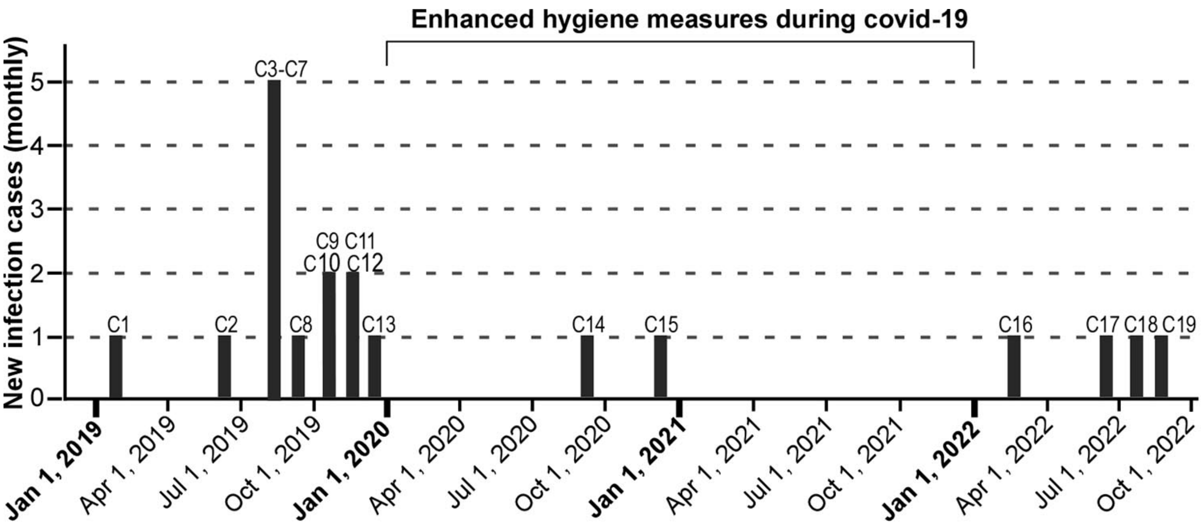

Appendix Figure 1. Monthly incidence of *C. vulturna* infections in a Shanxi, China hospital. C1–C19, patient cases 1 to 19. Only two cases of *C. vulturna* infections were found during the peak COVID-19 period, January 1, 2020–January 1, 2022, perhaps because of the enhanced hygiene measures implemented.

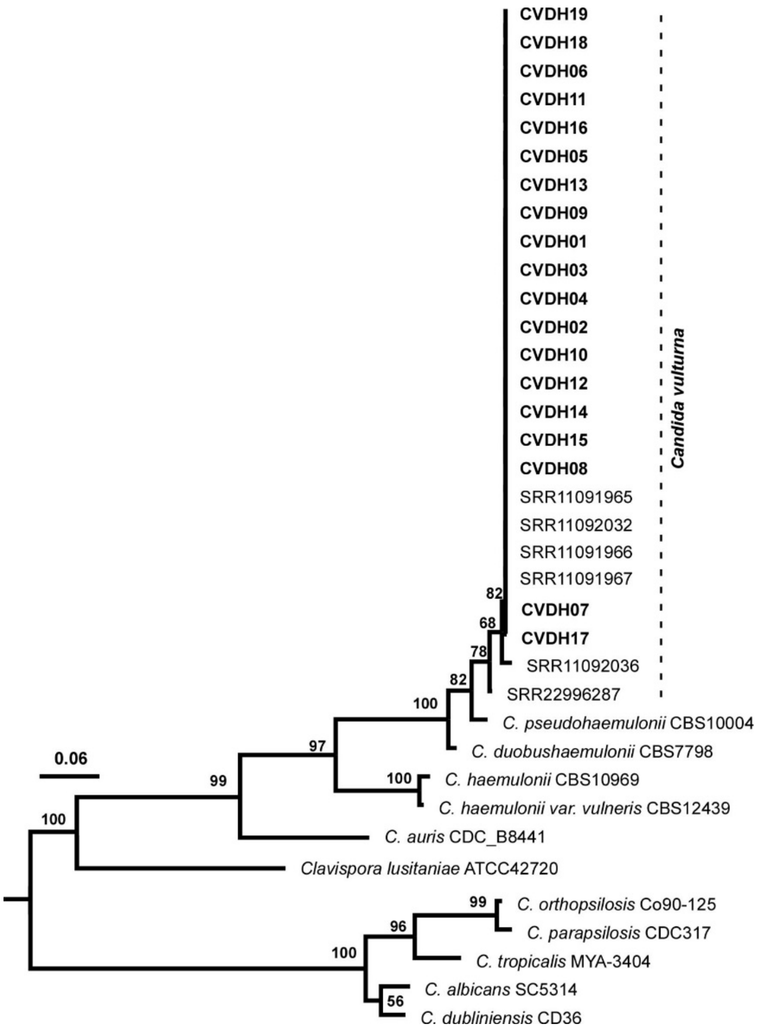

Appendix Figure 2. Maximum-likelihood phylogeny analysis of the CTG clade species based on the internal transcribed spacer and partial ribosomal sequences. The tree was generated using the program RAxML (https://cme.h-its.org/exelixis/web/software/raxml). The general time reversible model model, gamma distribution, 1,000 bootstraps, and midpoint root were adopted. Strain sequence information: *C. haemulonii* (CBS10969, JX459773.1), *C. haemulonii var. vulneris* (CBS12439, MK394151.1), *C. pseudohaemulonii* (CBS10004, MK394152.1), *C. duobushaemulonii* (CBS7798, MK394153.1) from the NCBI GeneBank; SRR11091965–67, SRR11092032, SRR11092036, and SRR22996287 from the NCBI WGS database; *C. auris* (B8441), *Clavispora lusitaniae* (ATCC42720), *C. parapsilosis* (CDC317), *C. orthopsilosis* (Co90–125), *C. tropicalis* (MYA3404), *C. albicans* (SC5314), *C. dubliniensis* (CD36) from the CTG database (http://www.candidagenome.org).

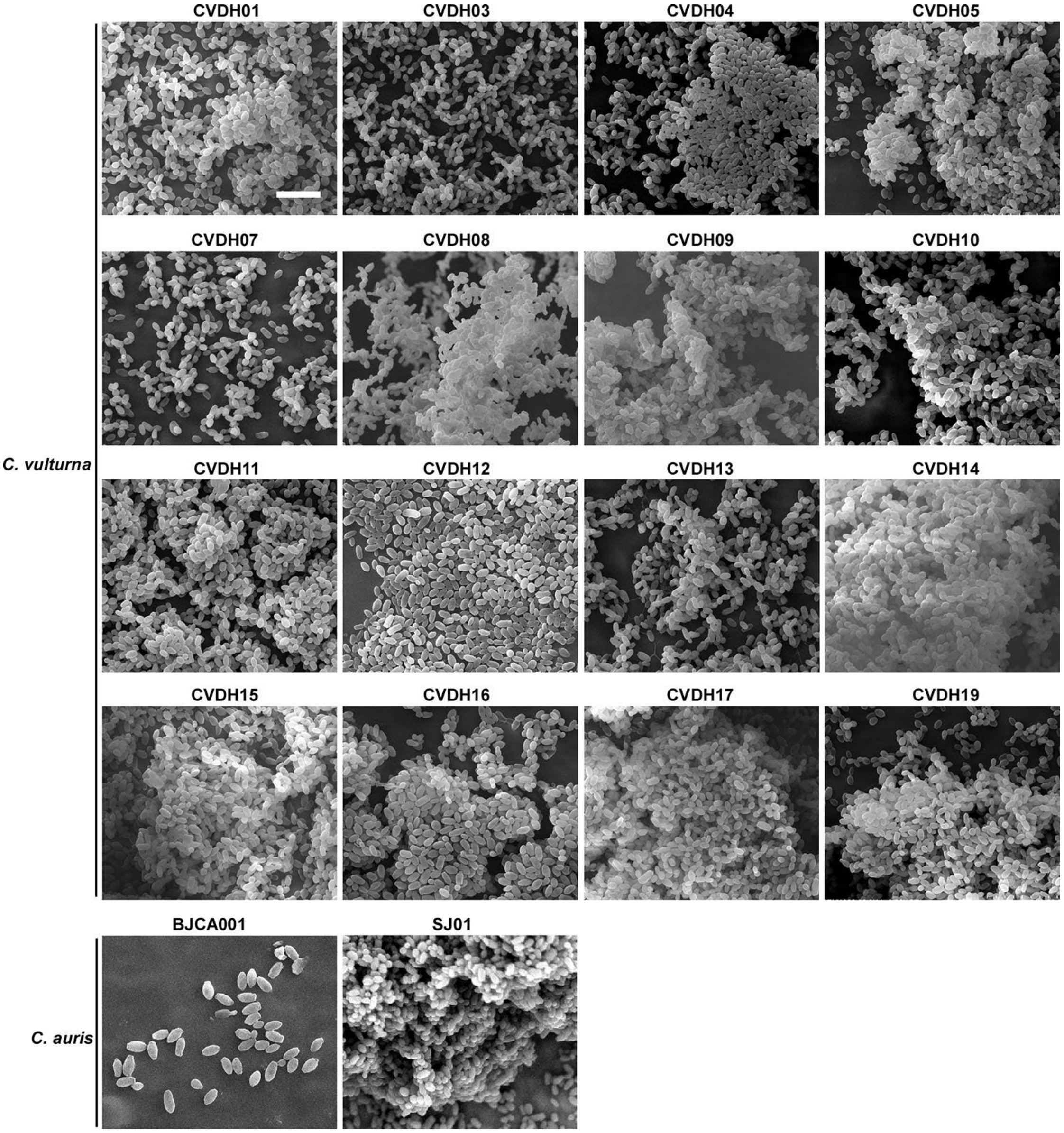

Appendix Figure 3. Biofilm morphologies of *C. vulturna* and *C. auris* isolates. *C. vulturna* strains are CVDH01, CVDH03–05, CVDH07–17, and CVDH19. *C. auris* strains are BJCA001 and SJ-01. Biofilms were developed on silicone squares at 30°C for 24 hours. Lee's glucose medium was used for biofilm growth. Compared to the other *C. vulturna* isolates, strains CVDH07, CVDH12, and CVDH13 developed weaker biofilms on the silicone squares. *C. auris* strain SJ01 developed robust biofilms, whereas *C. auris* strain BJCA001 formed comparatively weaker biofilms. This figure is associated with Figure 2 in the main article.
